# First‐Aid Practices and Knowledge Regarding Snake and Scorpion Bites Among Patients Attending Onandjokwe State Hospital, Namibia: A Hospital‐Based Cross‐Sectional Study

**DOI:** 10.1155/emmi/6658174

**Published:** 2026-04-11

**Authors:** Petrus Uushona, Albertina M. N. Shatri

**Affiliations:** ^1^ School of Medicine, Hage Geingob Campus, Bachelor of Medicine and Surgery, University of Namibia, Windhoek, Namibia, unam.edu.na; ^2^ Department of Human, Biology and Translational Medical Sciences, School of Medicine, Hage Geingob Campus, University of Namibia, Windhoek, Namibia, unam.edu.na

**Keywords:** first-aid measures, knowledge, patients, pre-hospital care, scorpion bite, snake bite, tourniquets

## Abstract

**Background:**

Snake and scorpion bites pose a significant public health burden, with an estimated global annual morbidity rate of 5.4 million and 1.2 million, respectively. Various first‐aid methods are commonly practiced in communities, aiming to improve the chances of survival after bites. This study evaluated community practices for first aid on snake and scorpion bites and examined the alignment of these practices with WHO recommendations. This will determine the need for targeted educational interventions to promote evidence‐based and safe first‐aid practices.

**Methods:**

A cross‐sectional study was conducted amongst patients served by the Onandjokwe State Hospital from November to December 2024, with a total of 103 participants included in the study. The study assessed practices on occasions of bites, the perceived need for intervention, health‐seeking behaviors, and barriers to healthcare access when bites occur.

**Results:**

A total of 103 participants were included in the study, with 36.9% of participants having personal experiences with bites and 74.8% knowing someone who has been bitten. Only 0.97% of participants have received formal first‐aid training for bites, and 91.26% indicated that their communities would benefit from educational campaigns aimed at imparting knowledge on appropriate first‐aid practices. Only 12.6% of participants reported recommended first‐aid practices. Potentially harmful practices mentioned included the application of tourniquets, making incisions at bite sites, suctioning venom from wounds, and consuming various substances such as urine, *Colophospermum mopane* leaves, and other unspecified traditional remedies. Participants exhibited positive health‐seeking behavior, with 98.1% stating they would go to the hospital after bites. This is important as it demonstrates continued public trust in the healthcare system, with individuals presenting to hospitals in cases of bites, where appropriate treatment, such as antivenoms, can be provided to them. However, several barriers, such as distance, finances, and transportation, were identified by participants as reasons that delay timely hospital presentation after bites.

**Conclusion:**

Communities would benefit from targeted educational campaigns on proper practices, and many would welcome such initiatives.

## 1. Introduction

Snake and scorpion bites are common occurrences affecting many people in different parts of the world, particularly those who reside in rural areas, resulting in large morbidity and mortality [1]. Epidemiological data revealed that more than 5.4 million cases of snakebites are reported globally per year, with around 81,410–137,880 deaths per year and many more reported morbidities from amputations and permanent disabilities [2]. The incidence of scorpion bites stands at approximately 1.2 million globally per annum [3]. Epidemiological data on the overall incidence of snake and scorpion bites in Namibia remain unavailable, despite the high prevalence reported in isolated studies [4], which highlights the need for studies on this prevalent matter. A study conducted at the Katutura State Hospital in Namibia reported 721 snakebites over one year (2015–2016) [4]. This demonstrates the common occurrences of bites in Namibia despite the scarcity of data. People most at risk of bites are those who reside in rural areas where healthcare facilities that are equipped with antivenom are not easily accessible, and thus “adequate first‐aid treatments are of the utmost importance in achieving a positive outcome on both mortality and morbidity” [5]. Venomous snakes affect victims through the different kinds of venom grouped as cytotoxic, neurotoxic, and hemotoxic [6] and result in local tissue damage as well as different systemic manifestations depending on the type of venom possessed. Scorpions mainly affect the nervous system, resulting in symptoms of hyperactivity, such as tachycardia, hypertension, cardiac arrhythmias, profuse sweating, and restlessness. [7].

Whilst the definitive management of venomous bites involves the administration of specific antivenom [8], appropriate first‐aid methods are beneficial in delaying the onset of toxicity [9]. According to the World Health Organization (WHO) [10], the recommended first aid after snake bites includes reassurance of the patient, immobilization of the affected limb, e.g., using a splint, putting the victim in recovery position, immediate transportation to the hospital, as well as application of pressure pads or bandage technique. Despite the above, however, there have been several first‐aid practices documented that are practiced by different communities that are found to be ineffective while potentially exposing the victim to possible harm [10–12]. Application of tourniquets is one of the most practiced first‐aid measures when venomous bites occur, with some studies finding that 95% of participants recognize this as an appropriate first‐aid measure [13]. It has, however, been shown that this method compromises blood supply to the extremity, resulting in necrosis and possible risk of amputation. This practice and others, such as traditional herbs/remedies, incisions at the wound site, electrocution, and ingestion of alcohol [12], are among the many harmful practices reported in the literature that result in increased morbidity and mortality amongst victims through delayed clinical presentation, inflicting potentially lethal injuries on patients as well as promoting local infections [11]. The WHO highly discourages most traditional practices and notes that “most traditional first‐aid methods should be discouraged: they do more harm than good” [10]. Data on the community first‐aid practices in Namibia for snake and scorpion bites remain unavailable and unstudied, despite the high prevalence of bites in our communities.

From the literature reviewed, it is apparent that despite the presence of evidence‐based practices, communities continue to practice their traditional first‐aid practices. Some of these practices have consistently been shown to be harmful in various literature reviewed here. There is a paucity of information on the epidemiological data on the occurrence of bites in Namibia, and further on the community practices of first aid when faced with potentially venomous bites, demonstrating the knowledge gap and need for research on this prevalent topic. The highlight of certain potentially harmful practices in the Namibian standard treatment guidelines [14] further alludes to the presence of these practices in our communities, although unstudied, thus necessitating investigation through research. Snake and scorpion bites are common occurrences and tend to affect people in rural areas more, where antivenom may not be readily available due to factors such as distance to hospitals and unavailability of transportation. [9].

Literature has identified many ineffective and potentially harmful first‐aid practices, such as the application of tourniquets, incision of the wound, electric shock application, and application and consumption of herbal remedies, that are commonly practiced in different parts of the world after potentially venomous bites. Data on the practices in Namibia, however, have not been researched, despite the high occurrence of bites as noted in some studies. There exists a knowledge gap in this critical topic in our communities. To decrease morbidity and mortality amongst bite victims that accompany potentially harmful first‐aid practices, research projects such as this must be carried out to identify the current practices in our societies and to determine the need for targeted educational interventions aimed at reducing ineffective and potentially harmful practices to overall improve patient outcomes and reduce morbidity and mortality.

This study is very significant because with the high prevalence of potentially venomous bites, patients often present with attempts having been performed to administer first aid, e.g., with a tight tourniquet, which, although intended to help the patient, is potentially harmful. These practices demonstrate a need for studies on community practices to identify potentially harmful practices, such as the application of tight tourniquets and incisions at the wound site, and to determine the need for corrective actions. Moreover, there exists a knowledge gap in the Namibian community’s practices on occasions of bites, and this project is a step in bridging this knowledge gap. The findings in this study would help gauge the knowledge, beliefs, and practices of community members after potentially venomous bites and subsequently help in determining the need for healthcare education to encourage evidence‐backed practices and discourage potentially harmful practices. Moreover, research on this very relevant topic opens an opportunity to identify novel practices or herbal medications that can be further studied and added to the arsenal of modern medications in treating bite victims when scientifically validated. The main aim of the study was to identify the first‐aid practices seen, known, or practiced by participants on occasions of snake or scorpion bites.

Therefore, the objective of this hospital‐based study was to assess the knowledge, beliefs, and first‐aid practices related to snake and scorpion envenomation among patients presenting to Onandjokwe State Hospital for various health concerns. In particular, the study aimed to document commonly used traditional and ethnomedical interventions and to explore how these practices align or diverge from WHO’s recommendations. By doing so, the study provides preliminary insights into patient behaviors and potential areas for public health education in a setting where data are extremely limited. We hypothesized that harmful traditional practices remain prevalent and that formal training is scarce in our setting.

## 2. Methodology

### 2.1. Research Design

This hospital‐based study was conducted from November to December 2024 using a cross‐sectional design that combined quantitative and qualitative approaches. Quantitative data were collected using a structured questionnaire. Additional open‐ended items were included to capture contextual information on traditional practices; these were analyzed descriptively and do not constitute a formal qualitative or mixed‐methods design.

### 2.2. Setting

The study was carried out at the Onandjokwe State Hospital, located in Oniipa, northern Namibia. The Hospital serves as the district referral hospital for the Onandjokwe District of the Oshikoto region of Namibia. The setting was chosen due to first‐hand observation of cases of bites by snakes and scorpions in the hospital, as well as due to the diversified population of patients from rural and urban areas that are seen at the hospital, which provides a representative sample for assessing participants’ knowledge and practices. The population studied was the community members who are served by the Onandjokwe Hospital, with participants selected from the pool of patients seeking various medical services at the hospital.

### 2.3. Sample and Sampling Method

The sampling was performed using a convenience sampling method of available participants who were willing to participate in the study. A total of 103 participants who met the inclusion criteria were willing to be interviewed for the study and were thus interviewed.

### 2.4. Inclusion and Exclusion Criteria

To be included in the study, participants needed to be chosen from (1) any of the patients seeking medical services at the hospital who were clinically stable and (2) those patients aged 18 and above and willing to take part in the study. Exclusion criteria included healthcare workers as well as patients below the age of 18.

### 2.5. Data Collection Tool

A validated 15‐question questionnaire was developed after reviewing literature sources to identify the various first‐aid practices that have been identified through similar studies in other locations. The questionnaire included the following sections:

1. Demographic information of the participants: Demographic data of participants were assessed in this section and included age, gender, educational level, employment status, and residence information.
2. Bite experiences: This section assessed the experience of bites amongst participants, assessing personal experiences with snake or scorpion bites as well as secondary experiences from people known to the participants
3. First aid knowledge: This section assessed information regarding various first‐aid methods that the participant knows of or has heard of, including things that can be performed locally to the bite site, as well as any items that can be consumed.
4. Health‐seeking behaviors: This section assessed the general health‐seeking behavior when bites occur, as well as factors that affect these health‐seeking behaviors
5. Need for education: This final section was an investigation of the perceived need for formal education, e.g., in the form of education campaigns on first aid amongst community members.

### 2.6. Data Collection Method/Procedure

Potential participants were contacted and screened to identify those who met the inclusion criteria for the study. The participant information leaflet (Appendix A) was presented to the patient to inform them about the nature of the study that is to be conducted. Participants who did not understand English had the leaflet verbally translated to them in their native language. Once the participants understood and were afforded a chance to ask any questions that they may have had, they were then presented with a consent form to sign. After consent was gained, data collection began in an interview style, whereby each question on the 15‐question questionnaire (Appendix B) was presented to the patient, and the response was recorded accordingly on the form by the interviewer. For those participants who were not eloquent in English, the questions were translated accordingly, verbally in their native languages, and the respective answers were subsequently recorded on the forms in English. There were no interviewer training procedures performed, as the researchers were the same individuals who conducted the study and therefore ensured that the questions were translated as intended to answer all the questions of the research project. Moreover, the researchers are fluent in English and the local languages.

### 2.7. Data Analysis

The data collected on physical copies of the questionnaire were entered into a digital version of the questionnaire using the Google Forms software to capture the data. This software allowed for the generation of a Microsoft Excel database of the responses. Data were cleaned using Microsoft Excel 2023, and Excel (2023) was used for thematic analysis of the qualitative open‐ended questions. In addition, the data were analyzed using SPSS software Version 30.0.0. Descriptive statistics such as percentages, frequencies, and means were used to analyze and describe the quantitative data, such as the demographic characteristics of the population, frequencies of various first‐aid practices, and health‐seeking behaviors. Tables and charts were generated to better display this information. All first‐aid practices mentioned by a participant were analyzed and grouped into 3 categories, namely, “recommended” for those respondents whose first‐aid practices mentioned fall in line with the recommendations by the WHO, “not recommended” for all the practices that do not fall into WHO‐recommended practices and lastly “mixed practices” for those responses that contain a mixture of WHO‐recommended and nonrecommended practices. This allowed frequencies of the various categories of practices to be quantified. For the open‐ended questions, thematic analysis was performed manually to group the various responses into themes/categories, with frequencies of responses falling under each theme being calculated, with examples of supporting quotes added.

### 2.8. Ethical Consideration

The research project was only conducted after receiving ethical approval from the University of Namibia’s Ethical Committee as well as the Ministry of Health and Social Services (Reference number: 22/4/2/3) (Appendix D: Approval letter). Patient data were only collected after the patient had received adequate information on the nature of the study, its aims and role as a participant, the voluntary nature of the study, and how confidentiality would be maintained. They were afforded the chance to ask questions to clear up any doubts. Only after the above were the participants allowed to permit data collection through the signing of an informed consent form (Appendix B). Data such as names were not collected to ensure that the participants’ identities remained anonymous. Data were stored in a safe folder as well as on an electronic database, which was under password protection for the duration of the research project, and which will be deleted after the completion of the research project. No harm or malicious intentions were intended or expected to come to the patient through this process.

### 2.9. Study Limitations

This study has some limitations, one of which is the hospital‐based design and use of convenience sampling, which poses the possibility of biases such as selection biases as well as limits the generalizability of the study to the whole population to some extent. In addition, data on bite experiences, first‐aid practices, and interventions were self‐reported and may be subject to recall bias. Participants attending a health facility may also differ from the general population in terms of health‐seeking behavior, which could influence their level of knowledge and reported practices. The lack of sample size calculation also poses some limitations to the study. Moreover, the questionnaire was administered in English and verbally translated into participants’ native language during interviews; while the researcher shared this language, the absence of formally validated translations may have introduced minor interviewer‐related variability. This study has a relatively small sample size (*N* = 103) and did not include a formal sample size calculation. As an exploratory cross‐sectional study using convenience sampling, the findings should be interpreted as preliminary insights rather than representative of the wider population. However, the study provides important initial information on community beliefs and first‐aid practices for snake and scorpion bites in a setting where published data are limited. Future research with larger, multisite community‐based samples is recommended to improve generalizability.

## 3. Results

### 3.1. Demographic Information

Table 1 summarizes the demographic data of the participants, with the highest age representative (24.3%) being 18–19 years old. The majority of participants are female, with the majority having had at least a secondary school level of education. The majority interviewed reside in rural areas.

**TABLE 1:** Distribution of participant demographic information frequencies and percentages.

Age	Frequency	Percentage (%)
18–29	25	24.3
30–39	20	19.4
40–49	12	11.7
50–59	19	18.4
60–69	11	10.7
70+	16	15.5

*Gender*		
Male	30	29.1
Female	73	70.9

*Education level*		
No formal education	17	16.5
Primary education	21	20.4
Secondary education	47	45.6
Tertiary education	18	17.5

*Employment status*		
Formal employment	31	30.1
Self‐employed	16	15.5
Unemployed	56	54.4

*Residence*		
Rural	72	69.9
Urban	31	30.1

### 3.2. Experiences With Bites

Exactly 38 participants (36.9%) have personal experiences of bites, with some having experienced snake bites, others having experienced scorpion bites, and one having experienced both bites personally. 74.8% of participants in total have second‐hand experiences with snake/scorpion bites from people that they know who have been affected. Table 2 summarizes the participants’ experiences with bites and breaks it down into each type of bite.

**TABLE 2:** Participants’ experience with bite frequencies and percentages.

Personal experience	Frequency	Percentage (%)
I have been bitten by a scorpion	30	29.1%
I have been bitten by a snake	7	6.8%
I have been bitten by both a snake and a scorpion	1	1.0%
I have never been bitten	65	63.1%

*Second-hand experiences*		
I know a victim of a scorpion bite	39	37.9%
I know a victim of a snake bite	24	23.3%
I know a victim of both a scorpion and a snake bite	5	4.9%
I know a victim of a scorpion bite, I know a victim of both a scorpion and a snake bite	2	1.9%
I do not know anyone	26	25.2%

### 3.3. Perceived Need for Information and Training Programs on Recommended Practices

**FIGURE 1:**
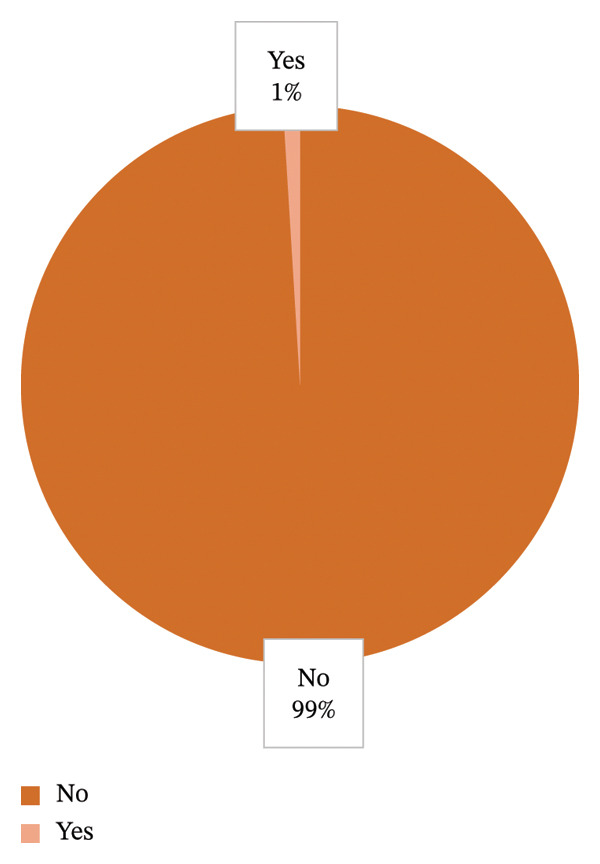
Graphical representation in percentage of level of formal training or information from official sources received by participant on recommended first‐aid practices when asked: “Have you ever received formal information/training on what to do after someone has sustained a snake/scorpion bite?”

**FIGURE 2:**
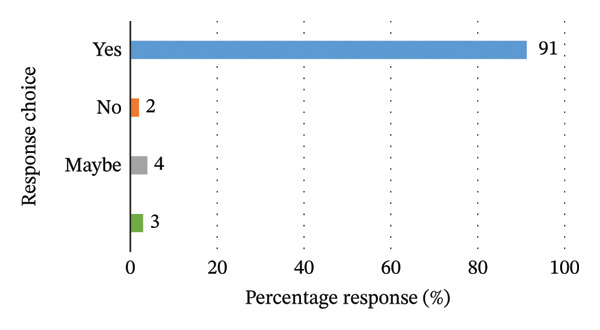
Graphical representation of desire for public training programs to impart knowledge on recommended practices when asked: “Would your community benefit from education on the right practices in cases of bites?”

Of all 103 participants, only 1 participant claims to have received formal training or information on the recommended first‐aid practices to offer to bite victims, whilst 102 deny having ever been trained or receiving such information from official sources. Most of the participants (94) welcomed the idea of educational campaigns and information sharing on recommended first‐aid practices, 4 participants chose the option that it could be beneficial (maybe), whilst 2 participants deemed it unnecessary for such campaigns. Figures 1 and 2 outline the level of training received amongst participants as well as the perceived need for educational campaigns, respectively.

### 3.4. First‐Aid Practices Employed by Community Members

**FIGURE 3:**
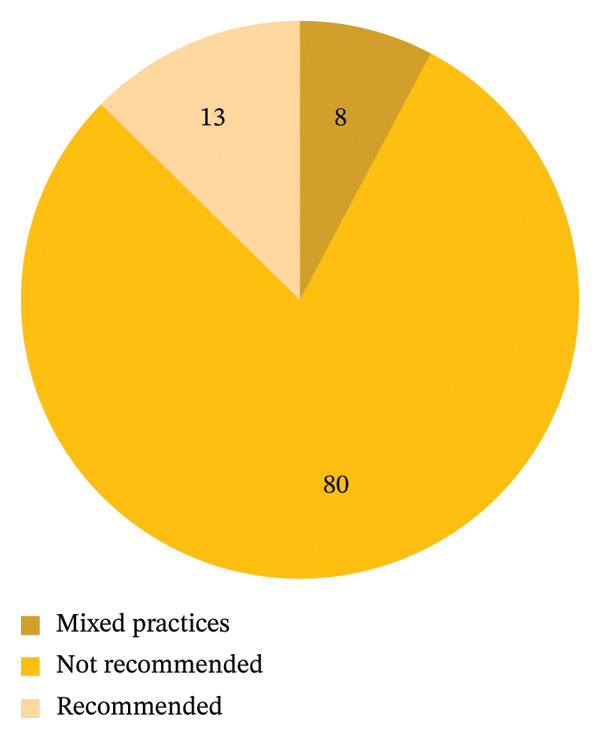
A graphical representation in percentages illustrating conclusions drawn from various practices mentioned, categorized according to WHO‐recommended first aid guidelines: as either “recommended” (aligning with WHO recommendations), “not‐recommended” (not based on WHO recommendations), and “mixed practices” (a combination of WHO‐recommended and nonrecommended practices chosen).

Practices mentioned were grouped into three categories, namely: “Recommended,” “not‐recommended,” and “mixed practices,” based on the WHO’s recommendations of appropriate practices of first aid following bites. Out of the 103 responses, 82 participants noted that they would perform practices that were categorized as “not recommended” (80%). Eight participants had responses that had a mixture of recommended practices as well as nonrecommended practices and thus coded as “mixed practices” (*n* = 8, 8%). Only 13 participants (*n* = 13, 13%) had practices that were in keeping with WHO “Recommended” practices (Figure 3). Specific practices mentioned by participants are summarized in Table 3, including the frequency of mentions of each practice.

**TABLE 3:** Frequencies of the various first‐aid practices mentioned and percentages out of 103 total responses.

First_aid_practice	Frequency of mentions	Percentage of mentions of practice per 103 total responses (%)
Make a cut/incision at the bite site	69	66.99
Apply tourniquet	53	51.46
Seek medical help immediately	20	19.42
Suck out the venom	19	18.45
Apply a traditional remedy to the wound	12	11.65
Seek a traditional healer immediately	7	6.80
Wash the bite site	4	3.88
Remove the victim from immediate danger	1	0.97
Squeezing out venomous blood	2	1.94
Others	13	12.62
Crushed/chewed Omusati/Mopane tree (*Colophospermum mopane*) leaves application	2	1.94
Unspecified tree leaves were applied to the wound	1	0.97
Apply the purple medication from the pharmacy	2	1.94
Apply a medication commonly referred to as another	2	1.94
Massage the wound area with warm water to prevent swelling	1	0.97
Burning at the wound side	1	0.97
Pour alcoholic beverages at the wound site	1	0.97
Take the snake to the hospital	1	0.97
Apply pressure to the area	1	0.97
Burn the snake and apply its ashes to the bite site	1	0.970,873,786

### 3.5. Items Consumed to Treat Bite Victims

Participants’ responses revealed a wide variety of scientifically unvalidated substances consumed as first aid treatment for bite victims. The most consumed substance was noted to be one’s own urine, which was mentioned by 23 participants, with some participants noting that it is used for scorpion bites (*n* = 7), others noting that it is used for snake bites (*n* = 1), and the majority noting that it can be consumed for both (*n* = 15). Sixteen participants mentioned the consumption of unspecified traditional remedies whose ingredients they are unaware of, whilst modern medicines such as painkillers were also noted to be consumed (10). Tree‐based remedies were utilized quite often, with the most mentioned tree being the Mopani tree. Other less commonly mentioned items consumed include raw onions, cooking oils, as well as a traditional medicine locally referred to as “othile” in Oshiwambo. Table 4 summarizes the responses to these items consumed as well as the frequency of mentions of each (Supporting material).

### 3.6. Health‐Seeking Behaviors and Factors Affecting the Health‐Seeking Behavior

The majority of the participants noted that they would go to the hospital after a bite (*n* = 101, 98.1%), whilst only two participants noted that they would not go (*n* = 2, 1.9%). Reasons attached to hospital attendance after a bite were categorized into various themes under which the various open‐ended responses were grouped as follows: “Believe in modern medicine,” “biting animals characteristic, e.g., size or type of animal,” “doctors expertise/skills,” “fear of death/adverse outcomes,” “first‐aid measures buy time to make it to the hospital,” “hospitals provide definitive/further treatment,” “traditional skills have been lost,” and “antivenom/venom neutralization.” The frequency of each response falling under the above themes as well as examples of quotes supporting each category are presented in Table 5 in the supporting materials.

### 3.7. Reasons Attributed to Delayed Presentation After Bites

Participants identified various factors that are associated with delayed clinical presentation to the hospital after bites. Lack of transportation was the most mentioned factor (43 mentions), followed by far distance to hospitals (28 mentions) and financial factors (26 mentions). Other, less frequently mentioned reasons for delays included the availability of ambulance services, fear of modern medicine, lack of knowledge of the danger of bites, and belief in traditional methods. Factors that contribute to delayed presentation are outlined in Table 6 under the supporting materials.

## 4. Discussion

According to the WHO, nearly one person is bitten by a snake globally every 10 s [15], whilst scorpion stings are said to be underreported at an estimated 1 million per annum globally because “many cases do not seek medical attention” [16]. Snake and scorpion bites are quite common, as demonstrated in this study, where many participants have either personal experience with bites (36.9%) or secondary experience with bites from occurrences amongst people that they know (74.8%). Despite the lack of epidemiological data in Namibia on the occurrence of snake or scorpion bites, the above results demonstrate that these incidences, although not officially studied, are quite high and highlight the need for further epidemiological data to truly characterize the burden of bites in Namibian communities.

The majority of the first‐aid practices reported in this study were not in keeping with the WHO’s recommendations for snake and scorpion bite management. The study identified practices such as making a cut/incision at the bite wound site (*n* = 69, 67%), application of tourniquet devices (*n* = 53, 51.5%), sucking out venom (*n* = 19, 18.45%), applications of traditional remedies to the wound (*n* = 14), and seeking of traditional healers (*n* = 7, 6.7%), which were commonly regarded as appropriate first‐aid practices by the participants of the study. These, however, are not in keeping with the WHO’s recommendations for first aid and are generally discouraged as they pose more harm than good. The WHO [17] recommends that, once someone is a victim of a snake bite, first aid should include immediately removing the victim from danger and detaching the snake if still attached to the victim, immobilizing the victim in a left lateral position to protect their airway, and providing reassurance. Further first‐aid measures include removing any occlusive items such as rings or bracelets, application of a pressure pad, as well as the utilization of the pressure immobilization bandage technique for neurotoxic snakes. Most of these practices remain absent in what the community deems appropriate as first aid.

Other studies evaluated on the topic show results similar to this study. A study conducted in Nigeria on prehospital practices in snake bites [18] noted that 74% of participants in that study chose application of tourniquets as appropriate first aid measures, whilst 11% chose to perform bite site incision, and 14% noted the ingestion or application of traditional remedies, whilst 4.2% chose suction of venom out of the wound. This study, however, mentioned the application of snake stones to the bite site, which was notably absent from all participant responses in our study [18]. Another study conducted in Morocco on healthcare workers’ experiences with scorpion bite victims’ prehospital practices noted similar practices to our study, with 90.48% of the participants noting that victims apply various variations of tourniquets after stings, 84.52% sucking out the venom, as well as application of various remedies performed by 64.29% of bite victims [19]. Burning at the site was mentioned by one participant, a practice also identified in a study conducted in an African setting [20]. A review article on harmful prehospital practices in the management of snakebites found that tourniquets were the most commonly used (45.8%), whilst other harmful practices included cuts to bite site (5.6%), ingestion of remedies such as various herbs (2.9%), as well as suction and application of product to the bite site [12], which were similar to practices outlined in our study.

Our study findings, aligned with findings of similar literature on the topic, demonstrate the high prevalence of nonrecommended and potentially dangerous practices. It further demonstrates the need for interventions to reduce possible morbidity and mortality among victims. The WHO, through its strategy to prevent and control snake envenoming, highlights the need to ensure safe and effective treatments as one of the key strategies to achieve the goal of reducing morbidity and mortality of snakebites by 50% before 2030 [21]. Through identifying practices such as these, stakeholders are equipped with information that can be used to put in place meaningful interventions that can be used to achieve this goal and improve health outcomes overall.

Of all the practices mentioned, only 1 participant mentioned that they would remove the victim from immediate danger, whilst 20 people mentioned that they would seek medical attention immediately. There were no mentions of other WHO‐recommended practices, such as splint immobilization of the affected limb, application of a pressure bandage, or reassurance of the patient as recommended by the WHO[10, 17]. Moreover, there was no mention of cold compresses and washing of the affected areas, which, together with the above practices, are recommended by the WHO for scorpion bites [22].

Several scientifically unvalidated substances have been mentioned by participants as being consumed as first aid for bites, as outlined in Table 5 of this study. The most identified remedies consumed for the management of bites are human urine (*n* = 23). Although novel sounding, the medicinal use of urine has been documented in many pieces of literature, with its consumption having been used for the treatment and prevention of certain bacterial and viral illnesses, as well as locally for wound cleaning [23]. Various studies conducted in Africa have, similarly to this study, reported the practice of urine consumption as first aid for bites [24, 25]. A study conducted in rural South Africa [24] reported that 29% of participants use their urine for the management of snakebites. Moreover, a qualitative research project conducted in rural Cameroon mentions the use of urine as first aid in managing snake bites [25]. No official literature, however, mentions or recommends the usage of urine for any medical purpose. Unspecified traditional remedies were mentioned by 15.5% of the participants for first aid, whilst cooking oil (0.97%), raw onions (0.97%), and a traditional substance referred to locally as “othile” (3.8%) are amongst the other specified traditional decoctions consumed.

Plant‐based regimens were reported in this study (9.7%), although most could not be identified by name. The Omusati/Mopani *(Colophospermum mopane)* tree was mentioned multiple times (*n* = 6, 5.8%), and it was noted that its leaves can be chewed to help with bites, whilst other participants mentioned the application of its leaves to the bite site. A study conducted in Namibia amongst Ovahimba community members found a wide medicinal usage of the Mopani tree, including management of stomach conditions, consumption of its leaves as an antiseptic for childbirth, as well as the usage of chewed‐up leaves applied to snakebite wounds [26], similar to the practice noted in this study. The medicinal usage of the Mopani tree has been reported for stomach pains, wound healing, remedies for gum bleeding, kidney stones, gastroenteritis, as well as impotence [27]. The extensive documented use of this tree in traditional medicines highlights the need for scientific research to identify and validate its medicinal properties. A review article conducted in Tanzania found that plant‐based regimens are quite common, with 109 different plants being identified as having been used for snake bite treatment [28]. The consumption of various concoctions and herbal remedies found in our study is consistent with the widespread usage of traditional remedies as documented in published articles and points to the need for further investigation of these widely used remedies to uncover potential medicinal compounds that can be integrated into modern medicine, as well as discourage potentially harmful herbs.

Most of the participants in this study (99.3%) have not received any formal training or information on first‐aid practices for snake or scorpion bite management. The WHO currently classifies snakebites as one of the neglected tropical conditions, and through its strategy for prevention and control of this disease burden, emphasizes improving community education regarding risk mitigation, the importance of seeking healthcare, as well as effective first‐aid measures as one of the key areas needed to improve control and prevent envenomation [21, 29].

This general lack of formal training or information on appropriate first aid found in our study aligns with similar studies in other Sub‐Saharan countries, demonstrating this general lack and need for professional information in our communities [21]. A study conducted in Malawi found a general lack of training amongst the study population on appropriate first‐aid measures for snake bites [21], similar to our results. Moreover, no evidence exists suggesting that any past mass campaigns to educate Namibians on the correct practices have been found. Participants, however, strongly agree with the concept of mass training programs/information campaigns to enhance the practices in the community, with only two of the participants noting that they do not think that the said training campaigns/programs are necessary.

The WHO emphasizes the importance of research to determine the “sociocultural, economic, political, and geophysical influence on perception of snakebites and treatment‐seeking” as a key pillar in its strategy for prevention and control of envenomation [29]. Participants in this study displayed positive health‐seeking behaviors, with the majority (98.1%) noting that they would go to the hospital after bites. Thematic analysis revealed that the reasons to seek medical help included believing that hospitals provide further/definitive treatment, understanding that first‐aid measures are temporary solutions that allow one to make it to the hospital, fearing adverse outcomes, as well as believing in modern medicine and the skills/expertise of healthcare workers. Reasons contributing to people not seeking care after bites were mostly the characteristics of the biting animal, e.g., people were less likely to present when bitten by a small scorpion vs. a large snake, and this highlights the need for information to dispel such myths. A potential limitation of this study is that it was conducted among patients attending the hospital for various health conditions, rather than in a community‐based field setting. However, it is important to note that participants were not seeking care for snake or scorpion bites, and therefore their responses reflect general health‐seeking behaviors rather than behaviors specific to venomous bites. While hospital‐based recruitment may not fully capture the health‐seeking practices of individuals who do not access formal healthcare, the findings highlight perceptions and needs among those who do seek care. In particular, the reported high demand for first‐aid training underscores an important area for public health intervention and education, even if community‐based studies are needed to provide a more comprehensive understanding of the wider population.

A study in Rwanda investigating health‐seeking behaviors amongst snake bite victims found that 87% of victims sought informal care, such as from traditional healers, whilst only 13% sought formal medical care immediately [14], which is inconsistent with the findings of our study in terms of health‐seeking behaviors as the majority of our participant note that they would go to the hospital even after initial first‐aid attempts. The main barriers identified to timely hospital presentation include transportation, distance to the hospital, as well as financial factors, and the belief in the usage of traditional factors. Less frequently identified barriers include lack of knowledge, e.g., on how serious the bite is; time of the day, e.g., at night; size of the animal; availability of ambulances; as well as fear of modern medicine. Similar studies in Rwanda identified distance to the hospital, lack of transportation, financial factors, and ambulance services amongst the barriers for timely access to healthcare services following bites [20], with results corresponding to the findings of this study. Most factors identified are modifiable and demonstrate the need for public health interventions to ensure access to adequate healthcare.

Models such as the health belief model are important in understanding why individuals adopt certain potentially detrimental behaviors concerning their health. In the case of this study, the continued usage of inappropriate first‐aid practices that are potentially harmful, even though most participants point out that they would eventually seek hospital care, suggests that individuals overvalue the importance of their own first‐aid measures and are willing to delay access to definitive care whilst partaking in these measures. Moreover, the perceived benefit of first aid far outweighs the perceived danger associated with these measures. These findings underscore the urgent need for targeted health education and intervention strategies that directly address prevalent misconceptions, while ensuring alignment with the Namibia Standard Treatment Guidelines [30], to promote evidence‐based, culturally appropriate care and improve patient outcomes.

## 5. Conclusion and Recommendations

The study identified a high prevalence of nonrecommended and potentially harmful first‐aid practices for snake and scorpion bites, highlighting significant gaps in knowledge and community vulnerability, both from harmful interventions and delays in seeking hospital care. Personal and second‐hand experiences with bites were common, indicating the need for adequate preparedness and community education to ensure timely and appropriate responses. While general health‐seeking behavior among participants was encouraging, several modifiable factors contributing to delays in care were identified and required targeted interventions. The study also revealed overwhelming support for formal training and awareness programs, emphasizing the importance of promoting WHO‐recommended practices. Future community‐based field studies across multiple regions of Namibia are needed to provide more representative population‐level data. Future studies should include multiple regions across Namibia to capture practices among diverse communities. In addition, locally mentioned remedies, such as Mopani tree−based treatments, were reported; although their safety and efficacy remain untested, they may warrant further research to assess potential medicinal properties and safe integration into evidence‐based interventions. Overall, community education, training, and targeted research are urgently needed to reduce morbidity and mortality from envenoming incidents in Namibia.

## Funding

The authors have nothing to report.

## Conflicts of Interest

The authors declare no conflicts of interest.

## Supporting Information

Appendix A is a participant’s information leaflet shared with participants to provide detailed information about the study’s purpose, participant roles, and contact details. Appendix B is the consent form that participants needed to sign to give ethical permission to join the study. Appendix C is a questionnaire containing 15 questions used to collect data for this study. Appendix D is the ethical approval letter from the Ministry of Health and Social Services authorizing the researchers to conduct the study. Appendix E is the supplementary data (Tables 4‐6) for the results section.

## Supporting information


**Supporting Information** Additional supporting information can be found online in the Supporting Information section.

## Data Availability

The data that support the findings of this study are available on request from the corresponding author. The data are not publicly available due to privacy or ethical restrictions.
