# Supplementary material for: First‐Aid Practices and Knowledge Regarding Snake and Scorpion Bites Among Patients Attending Onandjokwe State Hospital, Namibia: A Hospital‐Based Cross‐Sectional Study
Source: Emerg Med Int. 2026 Apr 11;2026:6658174. doi: 10.1155/emmi/6658174 (PMC13069967; doi:10.1155/emmi/6658174)
Supplement: Supplementary file 1 — Supporting Information Additional supporting information can be found online in the Supporting Information section. [file EMMI-2026-6658174-s001.docx]

**Appendices**

**Appendix A: Participants’ information leaflet**

**ASSESSING COMMUNITY FIRST AID PRACTICES DURING INCIDENTS OF SNAKE AND SCORPION BITES: A COMMUNITY-BASED SURVEY CONDUCTED AT ONANDJOKWE HOSPITAL**

**Dear Sir/Madam**

You are hereby invited to partake in this research study as titled above Conducted By Petrus Uushona, A medical student at the University of Namibia School of Medicine. This letter serves to provide information regarding the Nature of the study.

**Introduction and objectives**

Snake and scorpion bites occur commonly in Namibia as well as in other parts of the world. They affect people in rural areas most who may not always have timely access to healthcare services. First aid services are important in helping the victim of a bite when correctly practiced. There are however several first aid methods practiced commonly that are deemed to be potentially harmful to bite victims. This study aims to identify the commonly practiced first aid principles in the community in hopes of identifying any need for educational intervention to help communities practice safe and informed practices.

**Objectives**

The objectives of the study are as follows:

- To identify the first aid practices seen, known, or practiced by participants on occasions of snake or scorpion bites
- To Identify the reasons for seeking or avoiding modern medical attention on the occasion of snake or scorpion bites
- To ascertain whether the participants have previously been imparted with professional insights or training regarding correct first aid measures after bites.
- To determine the need for educational initiatives in communities regarding these first-aid practices

**Participant’s role in the study:**

Your role as a participant in this study would involve answering a few questions on your knowledge of or practices in first aid in cases of snake or scorpion bites. No personal information such as names or ID numbers will be taken that could identify the participant and thus this study is completely anonymous and voluntary.

**Who will see the data?**

The raw data will be analyzed by the researcher as well as be available to the research supervisor. This data will then be used to compute a research report that will be submitted to the university for grading. Additionally, data from this research will be presented at conferences and will be possibly published in Unam-recognized journals.

**Enquiries**

Researcher: Petrus Uushona: [petrus2521@gmail.com](mailto:petrus2521@gmail.com)

Supervisor: Dr Albertina Shatri: [aiikasha@unam.na](mailto:aiikasha@unam.na)

**Appendix B: Consent Form**

**ASSESSING COMMUNITY FIRST AID PRACTICES DURING INCIDENTS OF SNAKE AND SCORPION BITES: A COMMUNITY-BASED SURVEY CONDUCTED AT ONANDJOKWE HOSPITAL**

**Researcher:** Petrus Uushona: [petrus2521@gmail.com](mailto:petrus2521@gmail.com)

**Supervisor:** Dr Albertina Shatri: [aiikasha@unam.na](mailto:aiikasha@unam.na)

**Dear Sir/Madam**

This document serves as a consent form for data collection in this research project as titled above.

**By signing this consent form you agree that:**

1. The nature of this research has been explained to you
2. You Understand your role in this research project
3. You understand that the results will be published and shared
4. You agree to have your data collected
5. I hereby agree/do not agree to take part in this study

______________________________ ___________________

Signature of participant Date

______________________________ ___________________

Investigator Date Signature

**Appendix C: Data collection tool**

**ASSESSING COMMUNITY FIRST AID PRACTICES DURING INCIDENTS OF SNAKE AND SCORPION BITES: A COMMUNITY-BASED SURVEY**

**CONDUCTED AT ONANDJOKWE**

**HOSPITAL**

**Section 1: Demographic Data**

1. Age:

*Mark only one oval.*


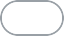
 18-29


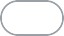
 30-39


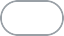
 40-49


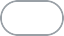
 50-59


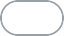
 60-69


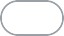
 70+

1. Gender:

*Mark only one oval.*


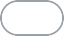
 Male
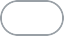
 Female

1. Educational level:

*Mark only one oval.*


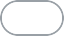
 no formal education
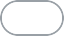
 Primary education
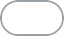
 Secondary education
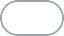
 Tertiary education

1. Employment status:

*Mark only one oval.*


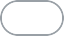
 Unemployed
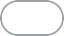
 Self employed


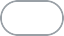
 Formal employment

1. Residence:

*Mark only one oval.*


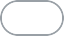
 Rural area


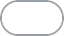
 Urban area

1. Have you ever been bitten by a snake/scorpion

*Mark only one oval.*


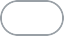
 I have been bitten by a snake


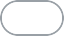
 I have been bitten by a scorpion


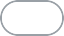
 I have never been bitten by both a snake and a scorpion


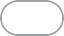
 I have never been bitten

1. Do you know someone who was bitten by a snake/scorpion

*Mark only one oval.*


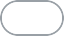
 I know a victim of a scorpion bite
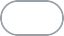
 I know a victim of a snake bite


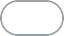
 I know a victim of both a scorpion and a snake bite


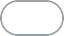
 I do not know anyone

*Skip to question 8*

**Section 2: Knowledge and practices of first aid in snake/Scorpion bites**

1. Have you ever Received formal Information/training on what to do after someone has sustained a snake/scorpion bite

*Mark only one oval.*

Yes

No

1. What have you heard of, seen or practiced as first aid measures after someone has sustained a snake or scorpion bite. (tick all appropriate)

*Mark only one oval.*

Apply tourniquet Suck out the venom

Make a cut/incision at the bite site

Wash the bite site

Seek medical help immediately

Seek traditional healer immediately

Apply traditional remedy to the wound

Remove the victim from immediate danger Immobilize the victim

Keep the bitten area below the level of the heart

Application of pressure bandage

Apply snake stones to the bite site

Other:

1. Have you heard of/ Seen or Practiced Consumption of anything after a snake or scorpion bites

*Mark only one oval.*

Alcohol

Traditional Remedies Modern medicines

Other:

1. Would you go to the hospital after a bite

*Mark only one oval.*


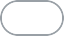
 yes
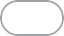
 no

1. Elaborate on the reason for your answer in question 11
2. What factors hinder people from bringing patients to the hospital in your community

*Mark only one oval.*

Transportation Financial factors

Believe in and usage of traditional factors

Lack of knowledge

Other:

1. Would your community benefit from education on the right practices in cases of bites

*Mark only one oval.*


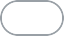
 yes


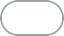
 Maybe


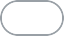
 No

This content is neither created nor endorsed by Google.

**Appendix D: Ethical Approval**


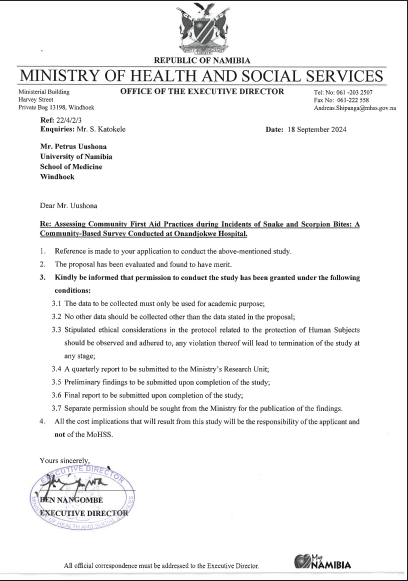


**Appendix E: Results: Tables 4-6**

**Supplementary materials**

**TABLE 4:** Mentions of Items consumed as first aid for bites

| Practice | Frequency | Percentage of mentions per 103 total responses (%) |
| --- | --- | --- |
| Urine Consumption (cumulative total) | 23 | 22.33 |
| for scorpion | 7 | 6.80 |
| For snake | 1 | 0.97 |
| for both | 15 | 14.5 |
| Traditional Remedies (Unspecified) | 16 | 15.53398 |
| Modern Medicines (e.g. pain killers) | 10 | 9.708738 |
| Tree/Plant-based Remedies (Cumulative total) | 10 | 9.708738 |
| Crushed roots of unspecified plants | 2 | 1.941748 |
| Unspecified Bushes | 1 | 0.970874 |
| Omusati/Mopani tree leaves | 6 | 5.825243 |
| Unspecified tree leaves | 1 | 0.970874 |
| Other specified traditional remedies (Cummulative total) | 6 | 5.825243 |
| Othile (local name) | 4 | 3.883495 |
| Cooking oil | 1 | 0.970874 |
| Raw onions | 1 | 0.970874 |

**TABLE 5:** Reasons for seeking hospitals

| **Theme** | **Frequency** | **Percentage (%)** | **Example quotes** |
| --- | --- | --- | --- |
| Believe in modern medicine | 15 | 14.6 | “to receive medicine available that is specific for that particular animal”  “hospital have medications to treat the poison” |
| Biting animal characteristics e.g. size, type (scorpion or snake) | 5 | 4.9 | “It is necessary to go there for snake bites”  “depends on how large the snake or scorpion is” |
| Doctors expertese/skills | 11 | 10.7 | “to get expert help”  “doctors have studied these animals and know more” |
| Fear of death/adverse outcomes | 12 | 11.7 | “to prevent death”  “bites are sometimes deadly”  “you might die if you do not go to the hospital” |
| First aid measures buy time to make it to the hospital | 17 | 16.5 | “you do not remove the poison but rather delay it”  “first aid methods give you time to get to the hospital” |
| Hospitals provide definitive/further treatment | 18 | 17.5 | “these methods do not remove all of the poison”  “to get further assistance” |
| No reason provided | 10 | 9.7 |  |
| Traditional skills have been lost with time | 6 | 5.8 | “people with skills to treat bites in the community aren’t as many as before”  “traditional skills have been lost” |
| Venom neutralisation/antivenom | 9 | 8.8 | “medication can prevent venom from going further”  “to remove the poison” |
| **Total** | **103** | **100.0** |  |

**TABLE 6**: factors affecting timely presentation to the hospital

| **Factors that hinder people from bringing patients to the hospital on time** | **Frequency of mentions of the factor** |
| --- | --- |
| Financial factors | 26 |
| Transportation | 43 |
| Believe in and usage of traditional factors | 18 |
| Lack of knowledge | 8 |
| Time of occurance e.g. Bites that occur at night | 5 |
| Far Distance to the hospital | 28 |
| Fear of modern medicine | 1 |
| Characteristics of the bitting animal e.g. size | 3 |
| No response | 2 |
| Availability of ambulance services | 2 |
